# Osteobiologies for Spinal Fusion from Biological Mechanisms to Clinical Applications: A Narrative Review

**DOI:** 10.3390/ijms242417365

**Published:** 2023-12-11

**Authors:** Byeong-Rak Keum, Hong Jin Kim, Gun-Hwa Kim, Dong-Gune Chang

**Affiliations:** 1 Research Center for Bioconvergence Analysis, Korea Basic Science Institute, Cheongju 28119, Republic of Korea; br0104@kbsi.re.kr; 2 Department of Orthopedic Surgery, Inje University Sanggye Paik Hospital, College of Medicine, Inje University, Seoul 01757, Republic of Korea; hongjin0925@naver.com

**Keywords:** spine, fusion, bone morphogenetic proteins, osteoblast, mesenchymal stem cells

## Abstract

Degenerative lumbar spinal disease (DLSD), including spondylolisthesis and spinal stenosis, is increasing due to the aging population. Along with the disease severity, lumbar interbody fusion (LIF) is a mainstay of surgical treatment through decompression, the restoration of intervertebral heights, and the stabilization of motion segments. Currently, pseudoarthrosis after LIF is an important and unsolved issue, which is closely related to osteobiologies. Of the many signaling pathways, the bone morphogenetic protein (BMP) signaling pathway contributes to osteoblast differentiation, which is generally regulated by SMAD proteins as common in the TGF-β superfamily. BMP-2 and -4 are also inter-connected with Wnt/β-catenin, Notch, and FGF signaling pathways. With the potent potential for osteoinduction in BMP-2 and -4, the combination of allogenous bone and recombinant human BMPs (rhBMPs) is currently an ideal fusion material, which has equalized or improved fusion rates compared to traditional materials. However, safety issues in the dosage of BMP remain, so overcoming current limitations will provide significant advancement in spine surgery. In the future, translational research and the application of clinical study will be important to overcome the current limitations of spinal surgery.

## 1. Introduction

Degenerative lumbar spinal disease (DLSD), one of the most common prevalent musculoskeletal disorders, is a leading cause of disability in the world [1,2]. Lower back pain, as the main symptom of DLSD, has been the greatest contributor to the Global Burden of Diseases, representing 7.7% of all years lived with disabilities [1]. In 2020, the prevalence of lower back pain was estimated to include more than half a billion people worldwide, which was expected to significantly increase by 2050 due to the aging population, especially in Asia and Africa [1]. DLSD encompasses any degenerative conditions in the lumbar spine, such as spondylolisthesis, disc degeneration, and spinal stenosis [3,4]. Among the various treatment options for DLSD, posterior lumbar interbody fusion (PLIF) is considered an effective surgical method to decompress the spinal canals, restore the intervertebral heights, and stabilize the painful motion segments [4]. With the surgical development of PLIF, minimally invasive techniques such as transforaminal lumbar interbody fusion (TLIF) and oblique lumbar interbody fusion (OLIF) have gained popularity, with the merits of early postoperative recovery and the reduction of surgery-related complications [5]. However, pseudarthrosis after lumbar interbody fusion is an important unsolved issue [3,6,7].

Achieving complete fusion in interbody segments is challenging for spine surgery [4,8]. While various techniques, including the addition of interbody cages and bone graft substitutes, contribute to achieving a high fusion rate, there are currently many limitations [5,9]. In this narrative review, we described the osteobiologies and current techniques for spinal fusion, from biological mechanisms to clinical applications.

## 2. The Mechanisms for Bone Regeneration

The bone regeneration process has historically been studied with the repair of fracture as a unique ability of our body by restoring it to its pre-injured functions [10]. In bone biology, homeostasis is regulated by two main cellular components: osteoblasts (bone-forming cells) and osteoclasts (bone-resorbing cells) [11]. Furthermore, various inflammatory cells and cytokines dynamically interact with these cells in bone environments, which are responsible for their repair capacity. For the bone regeneration process, previous studies have emphasized the role of osteoblasts with morphogen gradients such as bone morphogenetic proteins (BMPs) [8,10,12,13].

### 2.1. Bone Homeostasis

**Figure 1:**
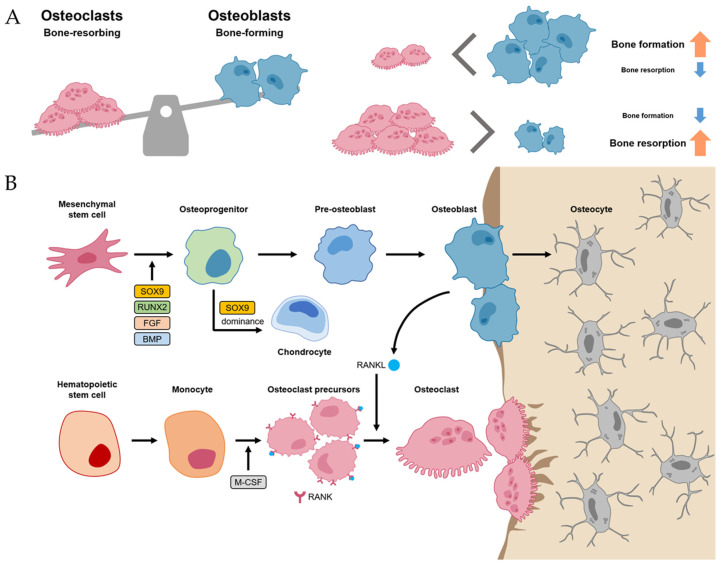
Bone homeostasis. (**A**) Normal bone homeostasis is balanced by osteoblasts and osteoclasts. (**B**) The detailed differentiation pathways of osteoblasts and osteoclasts in bone homeostasis.

Normal bone homeostasis is balanced by osteoblasts and osteoclasts (Figure 1A). Importantly, these two cells have different lineages between mesenchymal stem cells (MSCs) and hematopoietic stem cells (HSCs), respectively. MSCs, when induced by transcriptional factors, such as SRY-box transcriptional factor 9 (SOX9), Runt-related transcriptional factor 2 (RUNX2), fibroblast growth factor (FGF), and BMP, differentiate into osteoprogenitor and pre-osteoblast cells [10,14]. These cells, in turn, have the potential to differentiate further into osteoblasts, osteocytes, and chondrocytes, which are primarily involved in bone and cartilage formation. Of the many transcriptional factors, SOX9 and RUNX2 are the most important factors, and dominance between SOX9 and RUNX2 in MSC-derived progenitors determines their fates between chondrogenesis (SOX9 dominance) and osteogenesis (RUNX2 dominance) [15]. Osteoblast lineage cells not only differentiate into osteocytes but also promote bone mineralization by secreting hydroxyapatite and calcium (Figure 1B).

Meanwhile, osteoclasts, which have the function of bone resorption, are large multinucleated cells derived from HSC lineage [10]. Hematopoietic monocytes and macrophages were differentiated into osteoclast precursors mediated by macrophage colony-stimulating factor (M-CSF) [16]. M-CSF also proliferated the osteoclast precursors. Then, the osteoclast precursors differentiated into mature osteoclasts mediated by the receptor activator of nuclear factor kappa-B ligand (RANKL). Thus, M-CSF and RANK-RANKL signaling within bone environments is essential for osteoclastogenesis, which is initiated by the recruitment of osteoclast precursors by osteoblasts that express RANKL (Figure 1B) [10,17].

The homeostasis between osteoblasts and osteoclasts is very important in physiological bone environments [11]. If an imbalance between osteoclasts exists, it can develop into a pathological process such as osteoporosis (the environment for osteoclast activity surpasses osteoblast activity) or osteopetrosis (the environment for osteoblast activity surpasses osteoclast activity) [11,18].

### 2.2. Bone Regeneration Process

**Figure 2:**
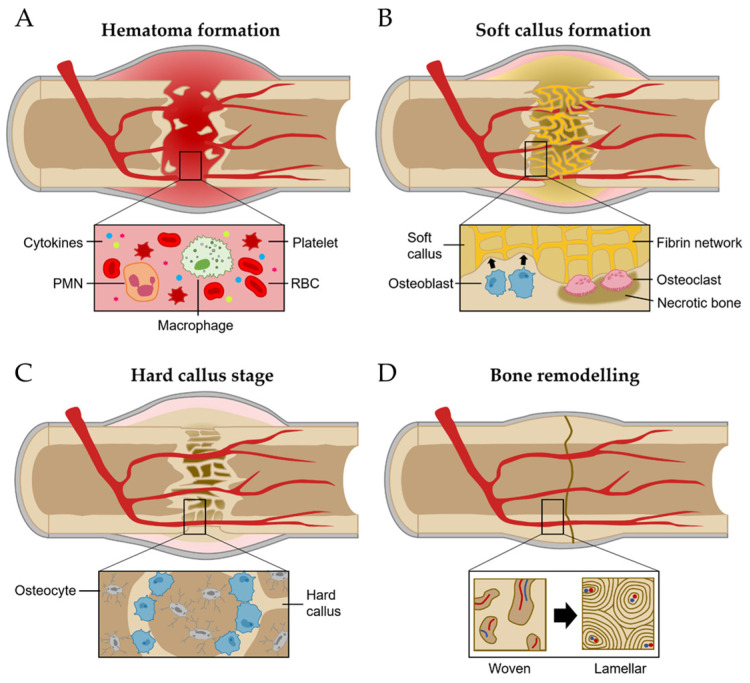
Bone regeneration process. (**A**) Hematoma formation. (**B**) Soft callus formation. (**C**) Hard callus formation. (**D**) Bone remodeling stage.

The regeneration process of bone has been studied with the fracture healing phase [19]. It involves a series of coordinated events, including hematoma-forming inflammation, soft callus formation, the hard callus stage, and bone remodeling (Figure 2) [8,20].

Inflammation stages occur for more than a few days after fracture [21]. The most important initial phase of the inflammation stages for bone regeneration is hematoma formation and inflammatory exudation from the injured blood vessels at the breakage location [21,22]. Injured soft tissues and degranulation of platelets release various cytokines, which results in typical inflammation responses such as vasodilation, hyperemia, polymorphonuclear neutrophils (PMNs), and macrophage migration and proliferation (Figure 2A) [19]. 

The network of fibrin and reticulin fibrils in hematoma is gradually substituted with granulation tissues (called soft calluses) and osteoclasts act to remove the necrotic bone at the fragment’s ends [19]. For soft callus formation, the osteoprogenitor cells in the cambium layer and endosteum are differentiated into osteoblasts, starting intramembranous appositional bone growth (Figure 2B) [10,19]. When the gaps between bones are linked with the soft callus, it starts to change the hard callus, called the hard callus stage [10]. For intramembranous bone formation, the soft callus within the gap is developed into the hard callus that is constituted with rigid calcified tissue by endochondral ossification [10]. Hard callus formation starts peripherally and progressively moves toward the center of the fracture and the fracture gap (Figure 2C) [10]. 

Following the solid union of gaps with woven bones, the remodeling stage begins [19]. The woven bone undergoes gradual replacement with lamellar bone, facilitated by surface erosion, condensation, and osteonal remodeling [19]. It persists from a few months to several years until the bone’s morphology is fully restored to its original state (Figure 2D) [19].

### 2.3. Signaling Pathways for Bone Formation

**Figure 3:**
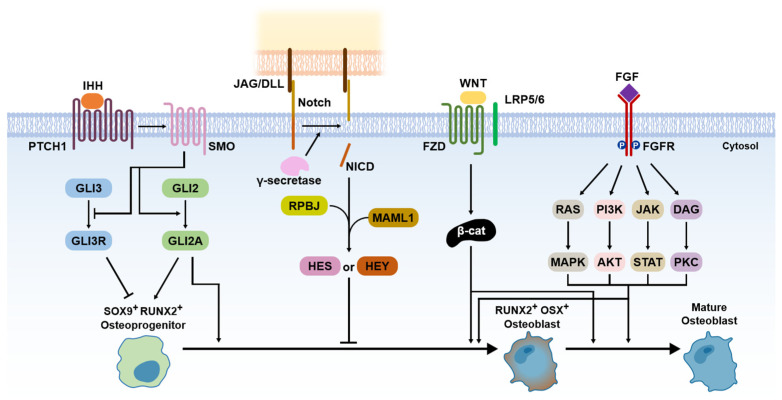
Signaling pathways for bone formation.

Since the osteoblasts have a critical function in bone formation, many studies have been focused on various osteoblast differentiation-related signaling pathways, which act in a coordinated manner to bone development and fracture repair [8,12,17,20]. In this review, we introduce the five representative signaling pathways, including Hedgehog, Notch, Wnt/β-catenin, FGF, and BMP signaling (Figure 3). Among them, BMP signaling is described in more detail in the next section.

Hedgehog has been known to be an important morphogen in limb development for the anteroposterior body axis [23]. The Hedgehog pathway also has a crucial role in endochondral ossification [10]. Indian hedgehog (IHH) acts with parathyroid hormone-related peptides in a negative feedback loop [24]. IHH binds smoothened homologue (SMO), which blocks the cleavage of GLI3 to the GLI3 repressor (GLI3R) and activates GLI2 to the GLI2 activator (GLI2A) [10]. It leads to the expression of SOX9 and RUNX2 in osteoprogenitor cells, which are master regulators for osteoblast differentiation [25].

Compared to other signaling pathways, Notch signaling is a negative regulator of osteoblast differentiation [10]. Notch receptors initially interact with Jagged (JAG) or Delta-like protein (DLL) families by direct cell-to-cell contact [10]. This leads to proteolytic cleavage of the γ-secretase complex, which releases Notch intracellular domain (NCID) [10]. The NCID interaction with RPBJ and Mastermind-like protein 1 (MAML1) induces the expression of Notch target genes, including Hairy and Enhancer of Split (HES) and HES-related with YRPW motif (HEY), which inhibits osteoblast differentiation [10].

Wnt/β-catenin signaling acts as a positive regulator of osteoblast differentiation [10]. The Wnt ligand binds Frizzled (FZD; a cell surface receptor) and low-density lipoprotein receptor-related protein 5 (LRP 5) or LRP6, which leads to β-catenin accumulation in the cytoplasm [26]. It allows for translocation to the nucleus expressing RUNX2 and osterix (OSX) [26]. The expression of RUNX2 and OSX enables the differentiation of osteoblasts [26]. The role of β-catenin is important for various signaling pathways for osteoblast differentiation because it stimulates transcriptional activity by interacting with the T cell factor (TCF)/lymphoid enhancing factor (LEF) [27]. Thus, the presence of β-catenin is also essential for osteoblast differentiation in the Hedgehog pathway [10].

FGFs are known as a family of signaling proteins that are crucial for normal development [8]. For osteoblast differentiation, FGF initially binds with the extracellular ligand-binding domain of the fibroblast growth factor receptor (FGFR), which leads to the phosphorylation of tyrosine kinase in the FGFR intracellular domain [10,28]. It activates the intracellular signaling cascade, including Ras/MAPK, PI3K/AKT, JAK/STAT, and protein kinase (PKC) pathways [10]. For fusion as a bone regeneration process, FGF2, among the more than 20 types of FGFs, has been shown to have superior osteogenic effects with a combination of BMP2 [8,10]. Thus, FGF2 is currently recognized as enhancing the BMP-induced bone regeneration process in the inflammatory and endochondral bone formation stages.

## 3. Bone Morphogenetic Proteins and Their Related Mechanisms

Since the discovery of BMP in 1958 by Marshall Urist, it has been well known as a potent inducer of bone formation [29]. BMPs are a member of the transforming growth factor (TGF)-β superfamily and researchers have identified more than 20 BMP lineages, with BMP-2, BMP-4, BMP-5, BMP-6, BMP-7, and BMP-9 recognized for their role in bone formation [13]. Since TGF-β not only recruits MSCs but also promotes the proliferation and differentiation of osteoprogenitor cells, the functions of BMPs are embryogenesis, cell differentiation, and skeletal development [10,13]. In particular, in bone regeneration, they also help with the endochondral bone formation stage [8]. The functions of the BMP subtypes are summarized in the next section. In this section, we describe the basic concepts of BMPs in the view of molecular and cellular pathways.

### 3.1. BMP Lineage

**Figure 4:**
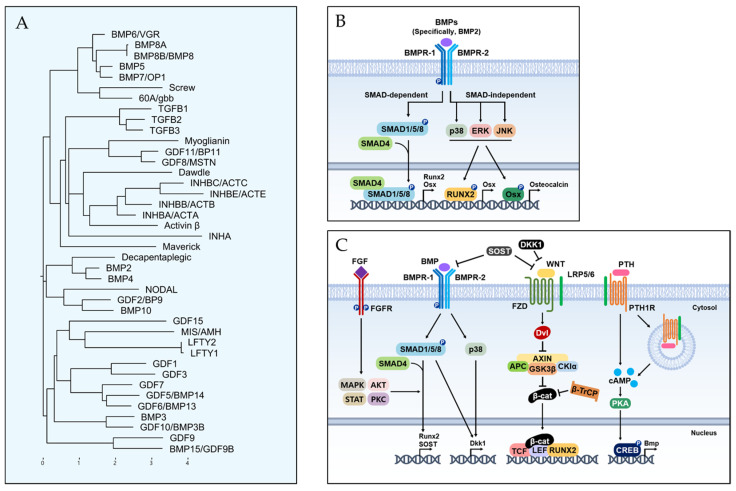
Bone morphogenetic proteins (BMPs) signaling pathways. (**A**) BMP lineage. (**B**) BMP signaling pathway for osteoblast differentiation. (**C**) Crosstalk between BMP and other signaling pathways.

It is historical to deal with BMP lineages and pathways because evolutionary records for multi-cellular animals have been suggested in many developmental processes of body axis determination [30]. Phylogenetic analysis provided the similarity in full-length protein sequences of human and fly orthologues (Figure 4A) [30,31]. Evolutionary conservation is notable for active mature signaling proteins after post-translational modifications of the pre- and pro-peptide domains [32]. 

### 3.2. BMP Signaling Pathway for Osteoblast Differentiation

BMP-2 and BMP-4 have representative functions on osteoblast differentiation, which are generally regulated by SMAD proteins as common in the TGF-β superfamily [13]. Thus, the current concepts of the BMP signaling pathway were divided into SMAD-dependent and SMAD-independent groups [8,10]. BMP signaling is initiated by the binding of BMP ligands to the BMP receptors (BMPRs) located in cell surfaces, which are transmembrane serine/threonine kinases [8]. There are two receptors in BMPR: Type 1 receptor (BMPR-1) and Type 2 receptor (BMPR-2). Upon ligand binding, such as BMP-2 or BMP-4, BMP ligands bring together and activate BMPR-1 and BMPR-2, forming a heteromeric receptor complex. BMPR-2 phosphorylates BMPR-1 and, subsequently, BMPR-1 phosphorylates SMAD1, SMAD5, and SMAD8 [8,10]. The phosphorylated SMADs form complexes with SMAD4, and the SMAD complexes translocate into the nucleus, where they activate the transcription of target genes such as RUNX2 and OSX [10]. The SMAD-independent pathway is mediated by the activation of BMP-BMPR via MAPK, ERK, and JNK (Figure 4B) [8].

### 3.3. Crosstalk between BMP and Other Signaling Pathways

For osteoblast differentiation, BMP has many important roles in many signaling pathways (Figure 4C) [13,17]. Firstly, the dual roles of BMP are well known for Wnt signaling. If BMP increases Dkk1 and SOST expression, it directly inhibits the Wnt signaling pathway, which leads to inhibiting β-catenin signaling [33]. BMP also promotes the Wnt/β-catenin signaling pathway, which is mediated by the formation of the β-catenin/TCF/LEF/RUNX2 complex from the antagonizing Dvl function [26,34]. Secondly, the BMP signaling pathway has a synergistic effect on osteoblast differentiation with the FGF signaling pathway [35]. Since FGF-2 is essential for nuclei translocation mediated by BMP signaling, the osteogenic effect of BMP-2 is enhanced with FGF [28]. Importantly, there were different characteristics between FGF-2 and BMP-2. For the bone formation process, FGF-2 was related to osteoblast proliferation, but it inhibits mineralization [36]. However, BMP-2 has a critical role in bone mineralization, which is caused by its involvement in different stages of osteoblast differentiation [10,13]. Third, in the crosstalk between BMP and Notch signaling, Notch signaling was negatively associated with BMP-induced osteoblast differentiation [13]. Furthermore, BMP-2 regulates the Notch pathway-related signaling pathway [37]. Lastly, PTH induces the differentiation of MSCs by enhancing the BMP signaling pathway by endocytosis of the LRP6/PTH1R complex (Figure 4C) [38].

## 4. Bone Graft Substitutes for Lumbar Interbody Fusions

The ideal bone replacement using bone graft substitutes is achieved by three properties in bone: osteoconduction, osteoinduction, and osteogenesis [4]. These three properties are essentially required for bone formation [20].

- Osteoconduction is defined as the physical property of scaffolding, which provides a microstructure to allow for bone ingrowth [39].
- Osteoinduction is defined as the ability to induce the production of osteoblasts, including substances and factors such as BMP [39].
- Osteogenesis is defined as a new bone formation cellular process from the differentiation of the osteoprogenitor cells [39].

These three factors are unique properties in bone regeneration compared to other scar tissues [39]. Therefore, in various fields, efforts to increase bone regenerative potential have been studied by utilizing these three properties [20]. Tissue-engineered products, such as nonbiodegradable polymeric implants, synthetic biodegradable polymer composites, graphene nanoparticles, and 3D-printed nanocomposite scaffolds, have been introduced but currently have limitations on clinical use [40,41,42]. In this section, we briefly describe common bone graft substitutes for spinal fusion, including autogenous bone graft substitutes (autografts), allogenous bone graft substitutes (allografts), growth-factor-based substitutes, and cell-based substitutes (Table 1).

### 4.1. Autogenous Bone Graft Materials (Autografts)

In spinal fusion surgery as well as osteosynthesis for non-union, the autogenous bone graft is considered the gold standard because autografts have all three properties (osteoconduction, osteoinduction, and osteogenesis) [4]. Autografts are obtained from various areas in spinal fusion surgery by a posterior approach. One method is to harvest local lamina bone obtained in the process of removing the bone for posterior decompression procedures [4]. These bones are likely to be accompanied by dead bone and/or severely degenerated bones, making them decrease the bone’s regenerative potential [43]. On the contrary, Park et al. clinically compared the union rates between autogenous ICBG and local bone grafts in spinal fusion up to three levels, which showed local bone grafts are also adequate options for lumbar spinal fusion surgeries [44]. Given the bone’s regenerative potential, harvesting the iliac crest bone by approaching the posterior superior iliac spine is the best option for autografts [4]. However, autogenous iliac crest bone grafts (ICBGs) have some limitations as follows. Additional incisions to harvest the ICBG lead to donor site morbidity (as reported up to 30%), which increases additional blood loss, operative time, and complaints of donor site pain [4]. With the procedure being prone to complications such as donor site pain, neuromas, and heterotopic ossifications after bone harvest, the use of ICBGs has currently been shown to have a declining trend with the development of other bone graft substitutes utilizing the principles of osteobiology [4]. Furthermore, local bone grafts are also clinically well-used in lumbar spinal fusion surgeries with other bone graft substitutes [45]. 

### 4.2. Allogenous Bone Graft Substitutes (Allografts)

Allografts were harvested from the cadavers so the amount of bone can readily be secured, which is one of the merits compared to autografts [9]. It acts as an osteoconductive potential that provides microstructures to regenerate the bone [20]. However, it has low osteoinductive potential, and the allogenous bone itself does not work for osteogenesis because there are no viable cells [20]. Despite the low potential of bone regeneration compared to autografts, allografts are widely applied in lumbar spinal surgeries and have been shown to have similar fusion rates and comparable clinical outcomes compared to the use of autografts in randomized controlled trials [46]. In the current status of posterior spinal fusion surgeries, allografts are contributed as autograft extenders because allografts are used with autogenous local bone (lamina bone), which has comparable outcomes with autogenous ICBGs only [39].

### 4.3. Demineralized Bone Matrix (DBM)

DBMs are medically approved biomaterials that an acid-extracted allogenous bone graft substitutes from the human bone resources, and are commercially available in variable forms from putty to gels [47]. They consist of type I collagen, non-collagenous proteins, and growth factors that contribute to osteoconductive and osteoinductive potentials but have a low level of osteoconductivity compared to mineralized allografts [47]. They have a variety of osteoinductive potentials because they contain low doses of BMPs (up to 0.1% by weight) [47]. With the osteoinductive potential of DBMs, some studies have reported increased fusion rates when supplementing the DBM with allografts in lumbar spinal fusion surgeries [48,49]. Thus, DBMs are currently used as common adjuvants for fusion materials.

### 4.4. Recombinant Human BMP (rhBMP)

After the elucidation of the role of BMP in endochondral bone formation, more than 20 subtypes of BMP have been studied and have various functions, not only in bone formation but also in the regulation of other tissues such as cartilages (Table 2) [12,26].

For these various subtypes of BMPs, rhBMP-2 and rhBMP-7 are clinically approved for use in humans [50]. BMP-2 and -7 are water-soluble and need a carrier to have an effective function during surgical procedures [13,50]. Carriers also have osteoconductive properties, so choosing the proper carrier can magnify the bone’s regenerative potential [8]. The use of rhBMP-7 (OP-1) is approved by the US Food and Drug Administration (FDA) for revisional spine fusion surgery under Humanitarian Device Exemptions but it has been withdrawn from the market [8]. rhBMP-2 was approved by the US FDA in 2002 for anterior lumbar interbody fusion (ALIF) with a titanium cage [4,8]. Many studies have demonstrated improved fusion rates by using rhBMP-2, which is more beneficial for complication rates compared to autogenous ICBGs [51]. Therefore, the off-label use of rhBMP-2 has been expanded from ALIF to PLIF to cervical fusion [4,8].

The disadvantages of the current use of BMPs are potential side effects despite higher fusion rates. BMP-related complications have been reported as inflammation, radiculopathy, heterotopic ossifications, osteoclast activation, urogenital events, and wound-related problems [52,53,54,55,56]. BMP-2 has preclinically demonstrated its induction of inflammatory cytokines such as IL-1β, IL-6, and IL-10 [56]. Inflammatory edema after use of rhBMP-2 can lead to catastrophic manifestations from serous formation to cervical spine swelling to airway obstruction to dysphagia [52]. Heterotopic ossification is also an important problem, which is caused by its potential for bone formation [51,53,56]. If it occurs near the nerve, radiculopathy or postoperative radiculitis may occur due to nerve compression. Interestingly, osteoclast activation by BMP-2 was preclinically illustrated, which was caused by the induction of RANKL and the repression of the Wnt signaling pathway [56]. Vertebral osteolysis and cage subsidence after the use of rhBMP-2 are thought to be the results of osteoclast activation by BMP-2 [53,56]. In addition, retrograde ejaculation and bladder retention were also reported after ALIF surgery, which was caused by mechanical or inflammatory injury to the superior hypogastric plexus [54,55]. Moreover, wound-related problems such as hematoma, wound dehiscence, and infection have been reported during the posterior approach from some pilot studies [56]. The mechanisms for high rates of wound-related problems have not been elucidated yet.

Some side effects (i.e., BMP-related complications) are significantly correlated with dosages of BMP, which were manifested in inflammatory edema, ectopic ossification, radiculitis, seroma, and vertebral osteolysis [50]. Specifically, in the case of cervical spinal fusion surgery using rhBMP-2, an inflammatory edema can lead to airway edema and dysphagia, which have devastating conditions [57]. Furthermore, rhBMP-2 was reported as leading to possible tumorigenesis despite low evidence in meta-analysis [58]. Spinal fusion surgeries need to have a higher volume of use of rhBMP-2 compared to other non-union surgeries, which can potentially lead to more complications [51,59]. Therefore, the combined use of rhBMP-2 and an autogenous local bone graft is one of the treatment choices to achieve comparable fusion rates and decrease potential side effects.

## 5. Current State and Future Aspects of Spinal Fusion

The current state of bone graft substitutes including BMP in spinal fusion are summarized in Table 3.

There were advantages and disadvantages in the selection of bone grafts. One of the advantages of LIF (such as DLIF, OLIF, and ALIF) from the anterior or lateral approach is direct access to the intervertebral discs [60,61]. This access made complete disc removal possible and allowed for more efficient placement of the bone graft substitutes with large-sized cages [61,62]. However, in the case of expected insufficient bone fusion, such as the use of the current DBM and allograft only, it is essential to posterior instrumentation to maintain spine stability [63]. Since many studies have currently provided the bone formative potential from BMP, the development of rhBMP is closely related to the safety and effectiveness of stand-alone LIFs in spine surgery [60,62,63]. In particular, in the usage of BMP, safety issues are accumulating in spinal fusion which are correlated with the dosage of BMP [64]. Interestingly, BMP-6 also has the ability to perform osteoblast differentiation like other BMPs (BMP-2, -7). Since it has about 20-fold higher infinity to BMPR than BMP-7, the clinical use of BMP-6 can significantly reduce the amount of BMP to achieve spinal fusion [12]. However, the development of BMP-6 is at the pre-clinical stage, so future trials should be needed.

Since MSCs can potentially differentiate into osteoblasts, they are a candidate for bone formation considering their osteogenic property [14]. Activated BMP signaling leads to the expression of osteogenic genes, especially Runx2, in MSCs. Runx2 orchestrates the expression of genes associated with osteoblast differentiation, such as alkaline phosphatase (ALP), osteopontin, osteocalcin, and collagen type I. Osteoblasts, differentiated from MSCs, produce extracellular matrix proteins and facilitate mineralization, forming hydroxyapatite crystals. As osteoblasts mature, they become embedded in the mineralized matrix and differentiate into osteocytes, contributing to bone remodeling and homeostasis. A phase I/II single-arm prospective clinical trial showed that 80% of 11 patients achieved one-level lumbar fusion with no adverse events with autologous MSCs, suggesting they are a feasible option in the future [65]. Gan et al. also tried posterior spinal fusions with enriched bone marrow-derived MSCs and porous β-tricalcium phosphate. They harvested enriched MSCs from bone marrow in the bilateral iliac crests perioperatively, with the combination of porous β-tricalcium phosphate granules [66]. In total, 95.1% of 41 cases had good union rates from the use of bone marrow-derived MSCs [66]. However, there are several challenges associated with MSC-based therapy. Firstly, MSCs obtained from different sources or even within the same source can display variations in their characteristics, potency, and differentiation potential, resulting in the limitation of the standardization [67]. Moreover, the potential for tumorigenicity, genetic instability, and unexpected side effects demand thorough investigation in preclinical and clinical studies for long-term safety issues [68]. Ongoing research has deepened our understanding of MSC biology, including its immunomodulatory mechanisms, paracrine signaling, and interactions with the host environment [69]. It is essential for optimizing therapeutic strategies. In conclusion, many studies are currently promising MSC-based therapy but several issues must be addressed through various clinical trials in the future.

## 6. Conclusions

With the development of mechanisms regarding osteobiologies, BMPs have been an essential factor in achieving spinal fusion. However, safety issues in the dosage of BMP remain, so overcoming current limitations will provide significant advancement in spine surgery. Furthermore, MSC-based therapy can be an alternative option to spinal fusion but great importance remains in the success of clinical studies. In the future, translational research and the application of clinical studies will be important in the advancement of spine surgery.

## Figures and Tables

**Table 1:** The properties of bone graft substitutes in fusion.

Class	Potential Properties		
	Osteogenic	Osteoconductive	Osteoinductive
Autograft (e.g., Cancellous bone of ICBG)	+++	+++	+++
Allograft (e.g., Freeze-dried bone, DBM)	−	+	+/− (allobone) ++ (DBM)
Growth-factor-based substitutes (e.g., rhBMP-7)	−	−	++

Autograft, autogenous bone graft substitutes; ICBG, iliac crest bone graft; Allograft, allogenous bone graft substitutes; DBM, Demineralized bone matrix; rhBMP, Recombinant human bone morphogenetic protein.

**Table 2:** The functions of BMP subtypes.

Class	Class	Functions
BMP-1	Metalloprotease	Regulation of the formation of the extracellular matrix (ECM) via acting on procollagen I, II, and III
BMP-2	TGP-β family	Key role in osteoblast differentiation, cartilage and bone morphogenesis, heart formation
BMP-3	TGP-β family	Ostegenin, inhibition of osteogenesis
BMP-4	TGP-β family	Regulation of the formation of teeth, limbs, and bone from mesoderms. An important role in fracture repair
BMP-5	TGP-β family	Cartilage development
BMP-6	TGP-β family	Osteoblast differentiation, chondrogenesis A role in joint integrity in adults
BMP-7	TGP-β family	Osteogenic protein-1 (OP-1), osteoblast differentiation, development of kidney and eye
BMP-8	TGP-β family	Osteogenic protein-1 (OP-1), bone and cartilage development

BMP, bone morphogenetic protein; TGF, transforming growth factor.

**Table 3:** Summary of bone graft substitutes in spinal fusion.

Class	Mechanisms	Efficacy	Limitations
ICBG	All capabilities	Traditional gold standard	Donor site morbidity
Allobone	Osteoconductive capabilities	Nearly not limited to the graft amounts	Infection risk of HBV or HCV
DBM	Osteoinductive and osteoconductive capabilities	Non-inferior to autoBG	Higher rates of spinal collapse
BMP	Stimulation of osteogenic differentiation of MSCs and new bone formation (i.e., osteoinduction)	Superior fusion rate in BMP/autoBG	Dosage and safety concerns
MSCs	Enhancement of spinal fusion by osteogenic effect	Not proven	Not proven

Autograft, autogenous bone graft substitutes; ICBG, iliac crest bone graft; Allograft, allogenous bone graft substitutes; DBM, Demineralized bone matrix; rhBMP, Recombinant human bone morphogenetic protein.

## Data Availability

Data are contained within the article.
